# Blockchain—Internet of Things Applications: Opportunities and Challenges for Industry 4.0 and Society 5.0

**DOI:** 10.3390/s23020947

**Published:** 2023-01-13

**Authors:** Amit Kumar Tyagi, Sathian Dananjayan, Deepshikha Agarwal, Hasmath Farhana Thariq Ahmed

**Affiliations:** 1 Department of Fashion Technology, National Institute of Fashion Technology, New Delhi 110016, Delhi, India; 2 School of Computer Science and Engineering, Vellore Institute of Technology, Chennai 600127, Tamil Nadu, India; 3 Department of Information Technology, IIIT, Lucknow 226002, Uttar Pradesh, India

**Keywords:** blockchain technology, Industry 4.0, Society 5.0, Internet of Things (IoT)

## Abstract

Today, blockchain is becoming more popular in academia and industry because it is a distributed, decentralised technology which is changing many industries in terms of security, building trust, etc. A few blockchain applications are banking, insurance, logistics, transportation, etc. Many insurance companies have been thinking about how blockchain could help them be more efficient. There is still a lot of hype about this immutable technology, even though it has not been utilised to its full potential. Insurers have to decide whether or not to use blockchain, just like many other businesses do. This technology keeps a distributed ledger on each blockchain node, making it more secure and transparent. The blockchain network can operate smart contracts and convince others to agree, so criminals cannot make mistakes. On another side, the Internet of Things (IoT) might make a real-time application work faster through its automation. With the integration of blockchain and IoT, there will always be a problem with technology regarding IoT devices and mining the blockchain. This paper gives a real-time view of blockchain—IoT-based applications for Industry 4.0 and Society 5.0. The last few sections discuss essential topics such as open issues, challenges, and research opportunities for future researchers to expand research in blockchain—IoT-based applications.

## 1. Introduction

A blockchain is a type of distributed ledger technology (DLT) that consists of a growing list of records, called blocks, that are securely linked together. Each node on the network has a copy of the same ledger in the blockchain. Each node also has its copy of the catalogue. It keeps track of how much money they spend together (i.e., messages sent from one node to another). A blockchain is where they are kept. They can be found and used at any time. They say blockchain and smart contracts will be the next big thing for a long time. People are paying more attention to them lately, which is why this is the case. As with the steam engine or the combustion engine, it could make many things in everyday and business life much better [1]. People are excited about the blockchain. Some people were worried about how much blockchain will be used worldwide in the next part. Because of these concerns, people think technology is underdeveloped and too exciting. When technology is used, the same results could have been achieved in other, more well-known ways. This technology’s power and efficiency make it exciting, smart, and robust. However, this often covers up the possible bottlenecks or adversities put forth by the same technology. As an alternative, it should be used to meet the needs of the people who use it. Many other insurance businesses began to look into how blockchain technology could be used in their industries/sectors [2].

Over a decade ago, blockchain was used for the first time. There is a shared database for transactions in a peer-to-peer network. This means everyone keeps all transactions in the same place [3]. Distributed computing might be a good solution for people who do not trust one person or group. They work together independently without needing to ask anyone else for help. During the early stages of bitcoin, Satoshi Nakamoto came up with the idea of money.

**Figure 1:**
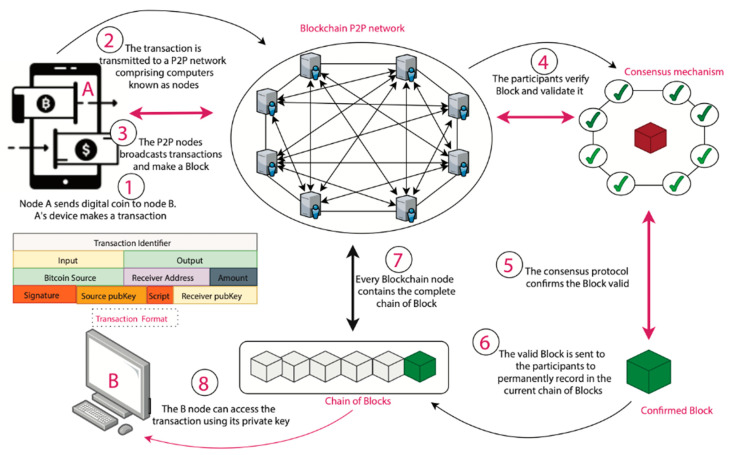
Processing of blockchain [3].

**Figure 2:**
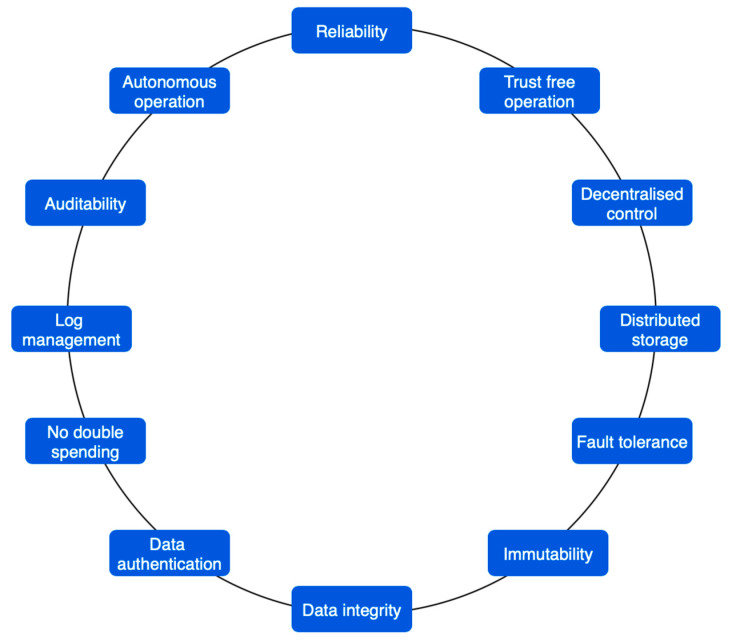
Characteristics of blockchain—IoT-based applications [4].

**Figure 3:**
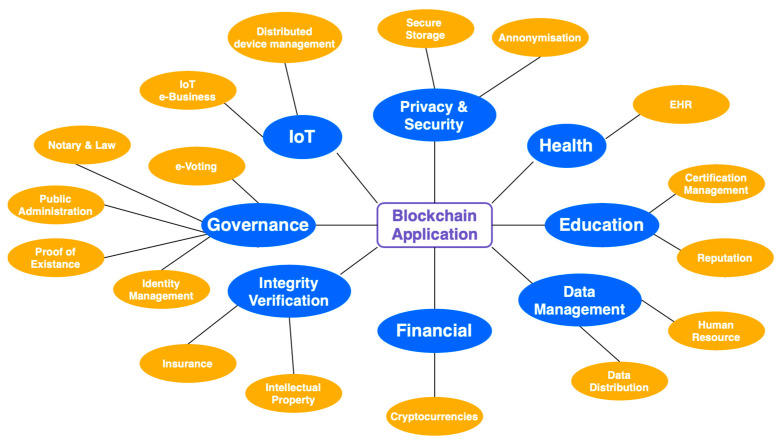
Blockchain-based applications.

Cryptocurrency: The first one used blockchain to build a system that everyone could use simultaneously. They wanted to make it easy for people to send and receive digital currencies, such as “bitcoins”, safely. This way, no one else had to give their approval for it. Unlike bitcoin, which does not have smart contracts (SC), this is different. Many people work together using traditional contracts and blockchain technology [4]. Figure 1 depicts the processing blockchain; whereas, Figure 2 explains several characteristics of blockchain—IoT-based applications. Many Internet of Things (IoT) applications rely on blockchain technology for security, privacy, and trust, as depicted in Figure 3.

However, “smart contracts” make it easy for agreements to be carried out in a shared environment if certain conditions are met, such as when they are signed. It was more accessible for people to create arrangements and trust relationships without writing them down in traditional contracts [5]. People cannot change the smart contract on the blockchain, so it cannot be changed. A lot of progress has been made in the last few years with smart contracts, but there are still many problems with how they work.

The issue of explainable AI is addressed by blockchain’s digital ledger, which provides information on the conceptual underpinnings of AI and the provenance of the data it uses. Artificial intelligence uses computers, data, and occasionally machines to simulate how the human mind makes decisions and solves problems. This increases confidence in the accuracy of the data and, consequently, in the AI’s recommendations. An audit trail is created when AI models are stored and distributed via blockchain, and combining blockchain with AI can improve data security.

This work is divided into seven sections: Section 1 describes the introduction to blockchain, whereas Section 2 describes related work. Section 3 describes artificial intelligence (AI)—Internet of Things (IoT)-based blockchain applications. Section 4 describes blockchain—Internet of Things-enabled environments, blockchain—IoT for intelligent agriculture, blockchain—IoT for the intelligent grid, blockchain—IoT for smart home and other appliances, blockchain—IoT for intelligent transportation, blockchain—IoT for banking and finance, blockchain—IoT for intelligent logistics and customer relationship management, blockchain—IoT for intelligent energy, blockchain—IoT for artificial intelligence-based security, blockchain Industry 4.0, and blockchain IoT for Society 5.0. Section 5 describes issues and challenges towards blockchain and blockchain—IoT-enabled applications/for the intelligent environment. Section 6 discusses open problems, challenges, and future research opportunities towards implementing blockchain in Industry 4.0 and Society 5.0 in detail. Further, Section 7 describes research statements for blockchain and IoT-enabled applications. At last, Section 8 describes the summary of this work in the form of a conclusion (including several interesting remarks for the future).

### 1.1. What Is a Smart Contract?

People use the term “smart contract” when they want an application to run business rules based on that business rules when things happen. This is called “automating business rules”, meaning that money or goods and services may be exchanged. Digital rights management content may be unlocked, and other data manipulations may be made to the data in the contract. A smart contract can only give people the information to do something [6]. People can create, share, manage, and keep up to date with the programmes that make up intelligent contracts in many ways. Blockchains are a way to store and exchange them. These technologies can hold and trade other types of digital money, such as bitcoin and Ethereum. People call them “smart contracts”. They are just words on paper that do not mean anything. Businesses use computer programmes to do, process, or make sales or purchases [7]. The execution must be tied to a legally binding agreement before doing this.

### 1.2. How Do Smart Contracts Work?

**Figure 4:**
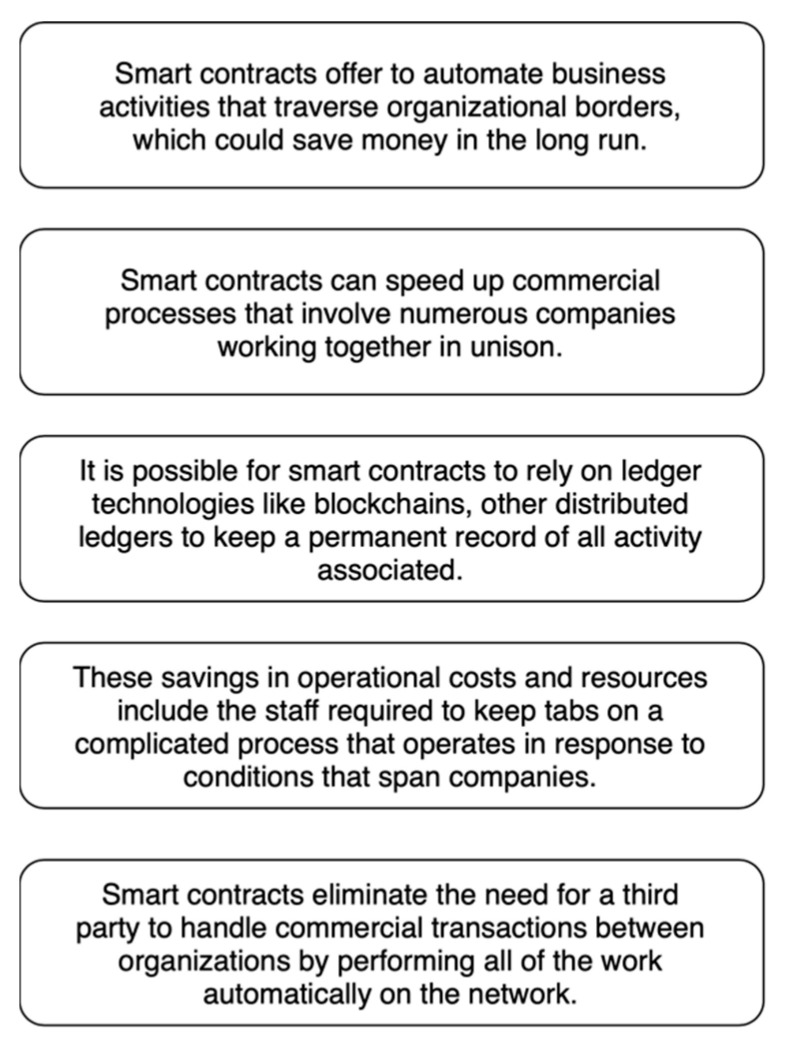
Benefits of Smart Contracts.

People use “distributed ledgers”, such as blockchains, to describe business rules that work on a virtual machine built into the blockchain. Smart contracts are used to describe these business rules and how they work. When business teams and programmers work together to figure out how their intelligent contracts should react to certain events or situations, the process of making intelligent contracts starts [8]. Figure 4 shows how smart contracts can help people.

Take simple things such as payment approval and delivery receipt into account in the future, which may not be possible now. More complicated things could be stored in it, such as the value of a financial instrument and how trades are made. There might be a payment from an insurance company if someone dies in a car accident or a disaster such as a flood. People do this use a lot. People talk about this when they do it, and it is called “encoding”. The people who make the platform then build and test it. This is why the application is sent to a different group. This way, the group will be able to protect it. This could be performed by someone at the company who knows about intelligent contracts or takes this kind of work [9]. It is used to start a contract after it has been agreed to. A “distributed ledger” is used to keep track of things. This is how the intelligent contract stays up to date: oracles, which are streams of data that feed the intellectual agreement with changes in events, do this. A cryptographic safety measure has made this source a safe place to visit. Oracles give the intelligent contract the right set of events, which works.

### 1.3. Future of Smart Contracts 

**Figure 5:**
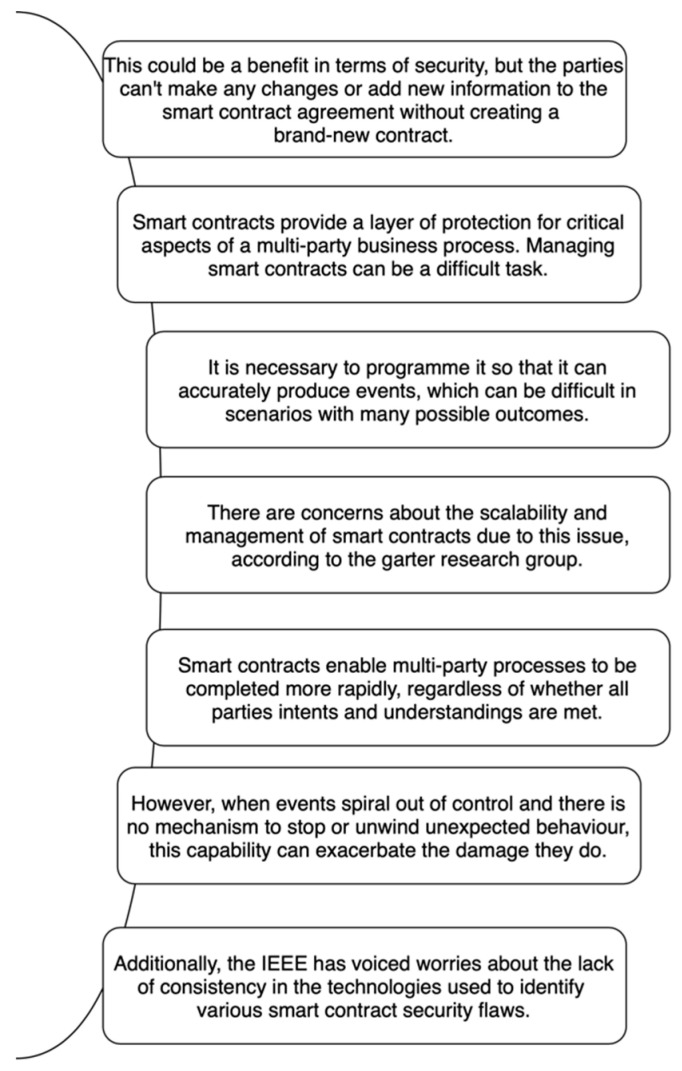
Common Issues and Challenges with Smart Contracts.

People do not understand intelligent contracts very well, but they can be used for much more than money. These people can conduct business in various fields, such as legal processes, insurance premiums, and crowdfunding agreements. They can also conduct business in financial derivatives and other areas. To cut down costs, smart contracts could make life easier and automate things that people now pay for. There may be a shift in the role of lawyers in the future as smart contracts become more powerful, and intelligent contract templates can be made to fit the needs of the people who use them. The people who work in a supply chain could have this happen. Using smart contracts can be challenging, as shown in Figure 5.

To make things even better, smart contracts can automate processes and set rules for people. When they have real-time checks and risk assessments, they can also help keep things safe. IoT and edge computing can also run smart contracts, which means they can ensure that things get completed. Suppose a utility company wants to create smart contracts to change electricity prices and work with devices built into power meters [10], this service is free for people who use electricity. For example, when prices reach a certain level, people who use intelligent contracts might have their air conditioners turned off or turned down. An IoT controller would turn them off or down in the next step. It also might be possible for people to buy things from vending machines with cryptocurrency. IoT could show that a smart contract might take money from a cargo container when it arrives. This is because the container has not been opened, and the contents have not been jostled too much as it has travelled across the country.

The main motivation of this paper is to analyse the use of blockchain when integrated with IoT and the possible use cases. Furthermore, this work highlights the important opportunities and challenges faced in technology and offers a larger scope for researchers to further elaborate on.

## 2. Related Work

Nick Szabo [10] came up with “smart contracts” in 1994. Szabo is a legal expert and a cryptographer who helped lay the groundwork for digital money. No digital platform or distributed ledger technology could help people make intelligent contracts until then. When the bitcoin cryptocurrency was created in 2008, it was made on a blockchain network with a distributed ledger that kept track of money transactions. This technology made it possible to write smart contract code that can put the terms of a contract into the blockchain.

Ghimire, Turusha, et al. [11] states that Industry 4.0 came about because people thought it would be better for manufacturing and the environment if they made things more modern and efficient. To help them make things, people use new technologies, such as the Internet of Things and robotics, to help them. These tools are used to make things. Many things can be performed with it, such as making it more secure and decentralised and showing how it works. A “blockchain” is how bitcoin works. People talked about it back in 2008. It has been a long time since there has been a lot of progress and change in how blockchain technology works. There are a lot of other businesses that use it, as well. These industries include healthcare and governance, the supply chain, entertainment, and many different things.

Carolina et al. [12] tell people that the main point of Society 5.0 is to use technology to solve problems such as ageing, low birth rates, and Japan’s lack of competitiveness. In this way, this is not the end: some other goals also exist. One of them is to help our country grow and build the foundations for a better world where technology does not leave anyone behind. They were made for this. The SDGs want to examine how people use modern technology and determine the best ways and tools for a constantly changing world.

Javaid, Mohd, et al. [13] argue that people should talk about how AI can help with the changes made in Industry 4.0. Many people work for businesses that want to make things work faster and cut costs. Some people want to be able to work with robots and people simultaneously. In innovative industries, machines that work together use AI automation systems to capture and interpret all kinds of data. These systems also help them work together. Systems like this make it easier for machines to work together, which is why they are essential. There are also systems like this one that make it easier for devices to work together. This is why they are crucial. In this case, many things talk to each other and work together. To make good decisions and figure out when things are not going right, businesses will use AI to process data from internet devices and machines linked together. In a business, it is easy for people to keep track of everything they do and how it goes.

Malau, Melinda et al. [14] argue that it is essential that the industrial revolution 4.0 changed how people work and live. Learning more about information technology makes the world more digital, so people become more aware of how it works. In the scientific field, this could not be good for people. It looked very different when the Industrial Revolution 4.0 came because tech progress seemed very different. It determined how accounting students learned in the fourth industrial revolution 4.0.

Guilherme F. et al. [15] have put forth a work with part one of Industry 5.0 called “Industry Strategy”. Part two was called “Innovation and Technologies”, and part three was called “Society and Sustainability”. Details of the industry called Industry 5.0 are made up of four components. Four elements make up the parts of the sector called Industry 5.0. If the Supply Chain 5.0 framework and its study plans follow this idea, they should be called Supply Chain 5. Industry 5.0 is still very early and ideal. Many new ideas and discoveries are found in the literature. There is not much of it yet, but there will be soon. People who study this kind of thing can also work on their projects. That means they can use it to conduct more work. It has a clear set of constructs and a well-organised research plan that they can use.

Chiehyeon Lim et al. [16] share a new way to think about how computer and industrial systems will work in the future, and businesses and people worldwide love it. The name for this idea is “industry 4.0”, and it is very different from anything else. There are still a lot that scientists do not know about this idea. There have been a lot of research and applications about Industry 4.0 that this work has looked at. It has found 31 things that need to be performed, and it needs to perform them. The next step is to figure out how to work together in Industry 4.0. This framework comprises six parts: connection, collection, communication, computation, control, and the creation of things. This study shows that experts’ ideas can be backed up using this data-driven approach. This indicates that this method is helpful and efficient.

Nivedita Haldar et al. [17] focus on the significance of how blockchain technology has become one of the most important and encouraging technologies in the world of Industry 4.0. Business and the economy are said to be changed by it. It has a lot of ways to help companies grow and start new ones, but it also has a lot of problems for traditional companies. This study wants to learn more about how blockchain technology can help run a business. A systematic literature review looks at how the apps work in different company parts. It also discusses the main problems with using blockchain and how it can help run a business.

Sá, Maria José, et al. [18] describe that digitalisation, the virtual world, and being constantly online are common today. People expect these things to become more critical in the future, but do not forget about those who are not a part of this reality (digital divide). People are now using the term “Society 5.0” to look at this process of shaping a society where the digital is used more and more for social and economic development is going on. This is called a “super-smart society”.

Marcello De Rosa et al. [19] explain how people are excited about the future of food production because of all the great things that can be performed with digital tools in farming. Some think that digital tools can help solve the problem of insufficient food. However, digitalisation has social, ethical, political, cultural, and environmental issues. A few people think using technology to make farming more environmentally friendly can help change. Even so, a trade-off may require more in-depth research into the many reasons for digitalising agriculture. Today, there is a need for science to show that digitalisation is good for the whole world. Finally, policies should make agricultural digitalisation accessible and open to everyone.

Yue, Kaifeng, et al. [20] use this text to explain how Trusted Third Parties (TTP) are often used in apps as a source of authority to make and check transactions. Customer convenience is essential, but the TTP paradigm has a lot of issues that cannot be avoided, such as security threats and privacy issues. Modern networks, such as 5G and beyond, do not always work best when using the TTP paradigm because it does not work for all of them. These networks have been getting better at supporting always-available, decentralised, and self-sufficient services in the last few years. From TTP-based to decentralised trust-based applications, people have significantly changed how people use them. It is because of what people think about blockchain technology and its use. Apps that use blockchains say there will be no trust in authorities, which will help with security and privacy. In blockchain research, the main goal is to look at ways to decentralise applications. This can lead to many new designs, from network architectures to business models.

Throughout this paper, we assess the state-of-the-art techniques related to the various aspects of blockchain and present our own opinions on the design of the solution, the research technique, and the research opportunities that the state-of-the-art now lacks. This is the research methodology that has been followed. Table 1, shown below, gives a comparison of the various works discussed in this section.

## 3. Artificial Intelligence (AI)—Internet of Things (IoT)-Based Blockchain Applications

There are three main parts to AI technology. They are data, an algorithm, and computing power. For algorithms to be trained, they need a lot of computing power. This means they need a lot of data to make a classification model. Sensors, Internet of Things (IoT) devices, and social media sites can all be used to acquire data in our significant data era. This also means that different people can own the same thing simultaneously. Many things could go wrong because of this. There are a lot of problems with having separate data sets [21]. People cannot use data from one source or stakeholder to train AI models. Either it is too expensive or too impractical to obtain a lot of data from all over the world and process it in one place. If one thing goes wrong, it could change the data, which could be harmful. Many different sources can make data from there look a little messy and vary in quality.

### 3.1. Data Sharing

AI needs a lot of information to be good at what it does. The more and better the data, the better the AI classification results. It is not easy to trust each other because the people who own the data for training do not trust each other. It is hard to sign off on or check the data. Then, there may be people who share malicious data for different reasons. This is the second reason. In the past, blockchain has been used to solve problems. This is not the first time that people do not trust a data market that is based on the blockchain. This platform lets IoT and AI solution providers talk and work openly. It allows people to sign up, search for data, buy, pay, and leave feedback through a smart contract. Using a Private Data Centre (PDC), users can keep an eye on what happens to their data to decide how to use them.

### 3.2. Preserving Privacy

Privacy is also a big deal regarding this, which is why when users share data, they will not keep their information safe. Obtaining personal information from a big company is terrible because they can use it. Deep LinQ is a distributed, multi-layer ledger that may keep data private when shared with others. Because it needs to be efficient, blockchain technology cannot store medical data. It could be used, but not because it must be completely decentralised. The branch layer is made to meet the needs of the people who use it.

### 3.3. Decentralised Intelligence

During the rapid growth of the IoT, much information about the IoT has been made. The results and models from a lot of IoT data can be found with the help of an AI service like Siri. There are so many IoT devices and edge computing devices out there. IoT and edge devices must share their data to make predictions and analyse what will happen next. People must start with this (such as intelligent monitoring, monitoring in other regions, and sharing data).

## 4. Blockchain—Internet of Things-Enabled Environments

People and businesses use a wide range of products and services thanks to the growth of telecommunication technology. How telecommunications services have changed affects many things, including aggregate measures, facsimile transmission, teleconferencing, computer-to-computer communications, SDN, e-healthcare, etc. Telecommunications Internet of Things (IoT) has also become a big market in the last few years. Satellite communications, 5G technology, and mobile communications are all intelligent communication systems that use this technology. Smart IoV is one of these systems. Communication and connectivity between these systems are better [22]. As the IoT market for telecommunications changes, more attention is being paid to how digital changes will happen. This can be performed with the help of telecommunications. There is a new way to connect smart devices to other things that do not need humans to work. This is called the Internet of Things (IoT). The Wireless Body Area Network is a group of IoT devices (also called intelligent devices). When people talk about this network, they say it is called the Wireless Body Area Network (WBAN). Two devices speak to each other, and the mobile device collects all the data they share. Then, the access points obtain the data from the mobile device, and the information is sent to the access points. In hospitals, it helps people tell people what to do and when. The data should not be given to anyone who could harm it while in transit to protect the company. This happens when sensors and actuators acquire data from many machines in an industrial IoT system. These data can make things more consistent and make the production process more efficient. It is also essential to ensure that the industrial IoT system is safe and can last long. IoT devices monitor traffic jams, air pollution, and security in an intelligent transportation application. This helps make the transportation system more efficient. This app has a problem regarding ensuring that the data it collects are safe. It is possible to control and change home appliances in a smart home app from afar with IoT-based smart homes.

Many people like it, but it also has a lot of security issues, such as confidentiality, integrity, and privacy. Public Internet is available between the intelligent devices and the gateway nodes near them to talk to each other. They send the data to edge servers close to them in an edge-based IoT environment. Most of the time, communication between intelligent devices, gateway nodes, and edge servers happens over public networks such as Wi-Fi. Several attacks, such as replay, man-in-the-middle, and privileged insider impersonation, can make this dangerous. Physical IoT device attacks can also happen [23]. A chain of blocks called a “blockchain” comprises other obstructions that could have information.

### 4.1. Blockchain—Internet of Things for Smart Agriculture

As many “nodes” make up the “blockchain,” each of these nodes has its own “distributed ledger.” Many nodes can access and update the same ledger simultaneously. They also share the work of keeping them up and running. The nodes use a “distributed ledger” in the blockchain to keep track of transactions between the nodes in a safe and long-term way [24]. It does not need third-party “intermediaries” because all the nodes share the databases, so they are not required. This means there is no longer a need for trusted third-party agents to verify, track, store, and sync transactions. Blockchain could keep the ledger’s data safe by moving the technology from centralised systems to decentralised systems and networks shared by many people. There are ways that blockchain technology can be used in business, but not all of them. That means they can be things such as food or raw materials. Music or data can also be digital, such as an app. All the group members write down what happened and can see the most recent ledger. People in a business network believe in each other when they see distributed intelligent ledger contracts and many agree. Once added to the list, removing it from the distributed ledger is impossible. Because it is encrypted, no one else can change it [25]. Because the blockchain cannot be changed and the distributed ledger cannot be messed with, the blockchain can be used as a source of truth and proof in the network.

### 4.2. Blockchain—Internet of Things for Smart Grid

Small businesses and people who used electricity for their homes could not afford to buy the equipment to make electricity. A customer brings the electricity into their home from a nearby power plant. They obtain electricity from the power grid and pay for it [26]. Even though new technology has changed how people acquire and distribute electricity, it has not been the same. Automation makes it easier to make a lot of things cheaper to produce. Sun, waves, geothermal, and other sources can now acquire electricity.

#### 4.2.1. Concept

People came up with a “blockchain” to solve the double-spending problem in digital money that did not have a central authority or spread power. When someone traditionally buys something, the money (paper or coins) is sent to the person getting paid. People use cash because it is made up of data that can be copied precisely and with little work in the digital world. In this case, the person spends both copies of the same money simultaneously. This is called “double-spending.” There must be trust between the two people to solve the problem. The intermediary is also needed to help solve a dispute between two people, which is why they are there. This is not going to happen if there is a dispute about the money. For example, this is not a good thing in the service business. Merchants will be wary of their customers because they are in this shape.

#### 4.2.2. Structure

**Figure 6:**
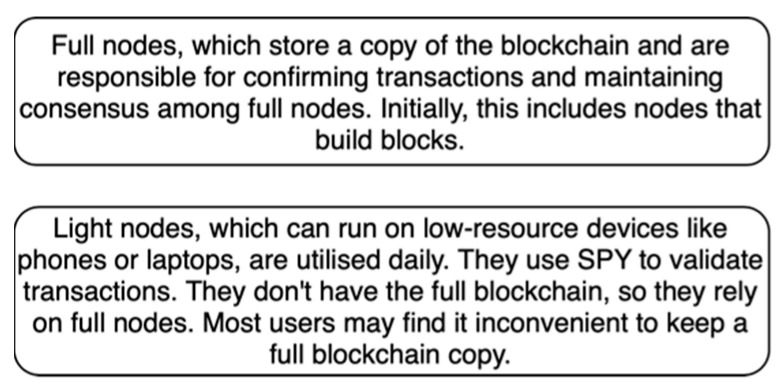
Types of Nodes.

A hash function links blocks together in a blockchain. In this way, each block is safe and related to the next one in a certain way. The league cannot be changed back because it was stamped correctly. They can give the money or the bitcoin to someone else if they own a certain amount. This is not true. As a replacement for a natural person, each owner is given a unique number called an address. A node is a person or thing that connects to a blockchain network and can perform many different things, such as send money. A node can join or leave the web at any time. It is like this: a tree has two main trunks. Shown in Figure 6 are different types of nodes.

#### 4.2.3. Ownership

**Figure 7:**
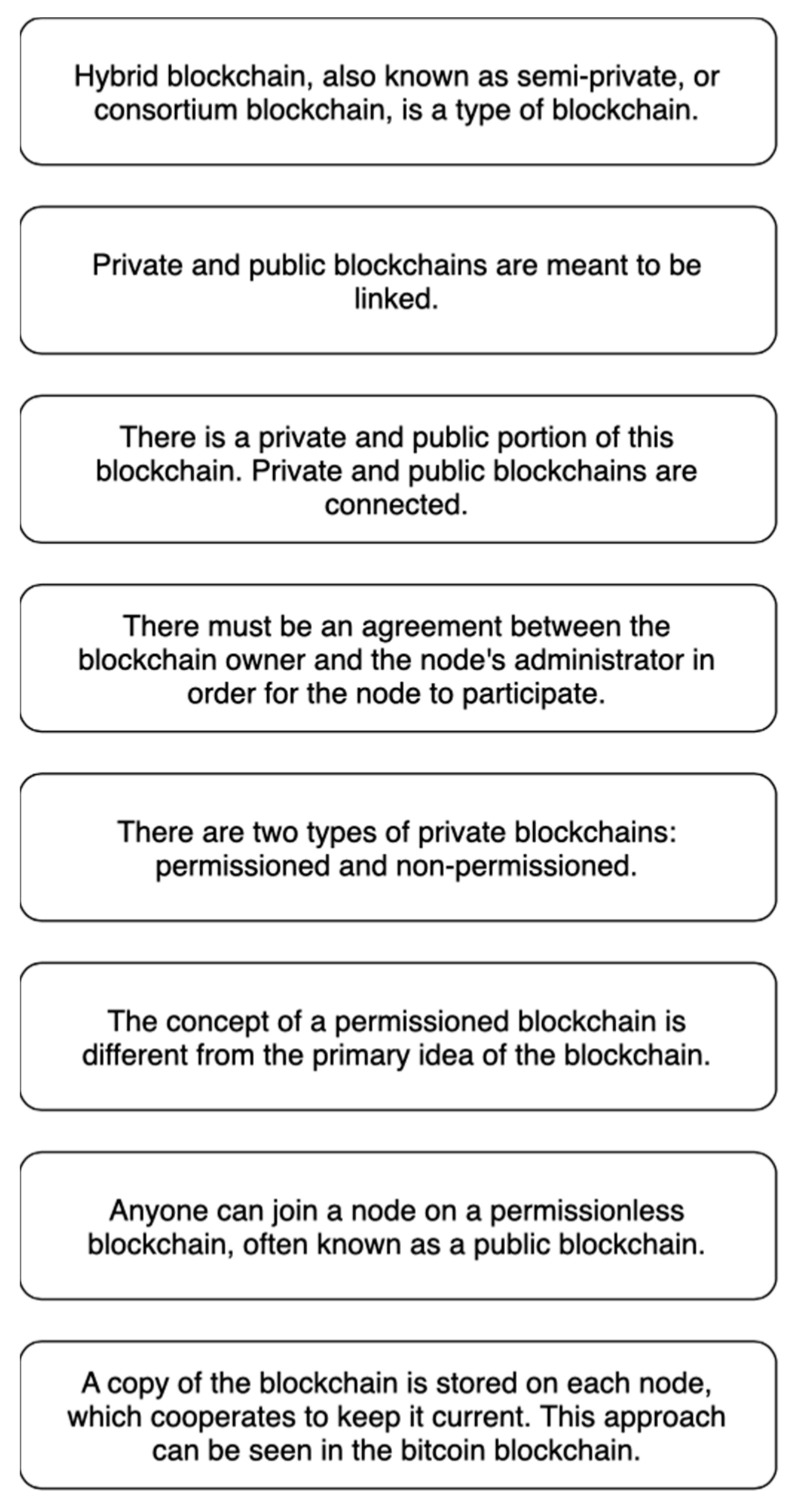
Blockchain Ownership.

This part talks about who owns what parts of the blockchain. This will decide who can be a node, check a transaction, and perform other things in the network. There are many ways to own a blockchain, as shown in Figure 7.

### 4.3. Blockchain—Internet of Things for Smart Home and Other Appliances

The Internet of Things (IoT) has been getting more attention recently because more and more devices are embedded in our daily lives. Though this growth looks good economically, people wonder how safe the country will be in the long run [27]. It is better to give each node more power than depend on another person or server.

The three main parts are IoT, smart home, and blockchain. The Internet of Things makes it possible for mobile devices to connect to the Internet and be controlled from afar with the help of the IoT. As a bonus, they have a lot of different sensors that can tell us what is going on, too. There are many smart gadgets used in the smart home environment. Among these are our TV and washing machine, air conditioning, lights, and other things. This way, people can watch their homes and appliances without risking their safety. When there was an IoT network, there would be a lot of data moving between these different things. All the time, they talk about important things. Humans do not play a role in this, which is a good thing. They share information all the time. This is how it works (a database, either in a server or a cloud). People can use the Internet of Things to send data to private blockchain ledgers in tamper-proof transactions that others can see. This way, no one else can change the data. People can also get in and out through IoT and the blockchain network. Most of this paper talks about how to use smart contracts in a blockchain-based IoT architecture to ensure that the security and privacy of the devices are light and decentralised so that they do not have to work together to keep them safe. Mainly, this paper shows that two or more people agree to do something for each other in writing.

Ethereum is a decentralised, open-source blockchain with smart contract functionality. It can be used to make things happen on the web. As a platform, Ethereum is used to create smart contracts that work. How it works: Ethereum is the best blockchain platform. In a smart home, many people want to use the same things and services that they do now. These all connect to a single IoT hub, which ties them all together; the government must approve each machine before getting any money from the government. Each transaction should be added to a blockchain as soon as possible so everyone can see it. There needs to be a smart home IoT hub set up so tokens can be stored for use.

### 4.4. Blockchain—Internet of Things for Smart Transportation

In the last few decades, many people have moved to cities because they have better jobs and a better education system. Better services, transportation, and communication have made cities better for living in [28]. It is the goal of smart cities to make people’s lives and living conditions better by using the Internet and modern technology to solve problems in cities. Smart cities use data from many sensors to figure out what is happening at different times and places. Work is going on worldwide to make a connection, and a well-equipped city is called a “smart city”. Using cloud computing, Industry 4.0, and the Internet of Things (IoT), the smart city uses these new technologies. This could cause problems with smart city data, services, and apps.

For this reason, people are worried that their data are not safe or private and that it can be seen by anyone. People need to believe in intelligent cities if they want them to be open and participate in the business, trade, and economic growth in order to improve living standards and obtain essential services. To work and move around the city, people need transportation. There needs to be an intelligent transportation system for people who live in today’s cities. This makes it easier for tourists and residents to enjoy their time in the city more comfortably and with more fun. They also help people build a collaborative ecosystem and improve the world.

In each block, information or a transaction has been time-stamped. In this case, these blocks cannot be changed. Because of this, the blockchain is a safe and trustworthy place for people to work. There are not many consensus algorithms that can be used on computers that do not have much power. Mining gives each node a share of the work that goes into making a choice. Mines use a lot of computer power [29]. The old security and privacy methods do not work well for intelligent transportation systems. Some problems and limitations still make it hard for blockchain technology to be used in many fields. Blockchain technology has clear advantages and opportunities to improve efficiency and cut costs, but there are still some problems and limitations.

**Figure 8:**
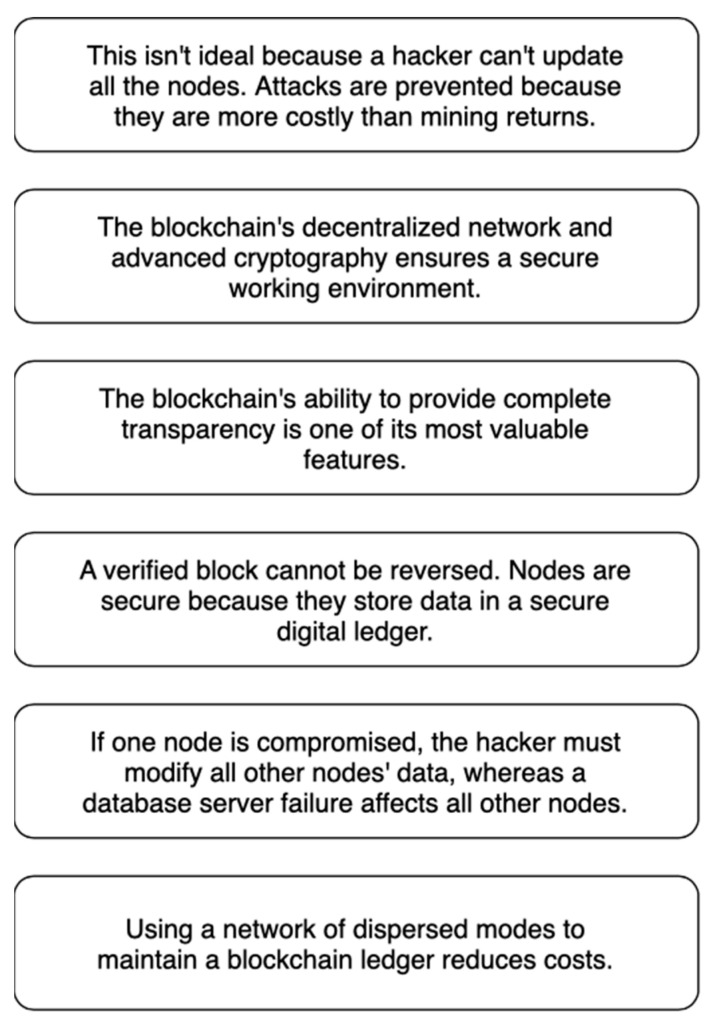
Blockchain—IoT for Smart Transportation.

Another thing to remember is that blockchain platforms should either wholly or partly replace systems that take a lot of time and money to use. DLT has been used in communication systems that can immediately impact big data. It also needs separate accounts to track how much data are being made. Because of this, there needs to be a new or specific blockchain app to deal with this issue. This is why an autonomous, connected, electric, shared dock-less vehicle system has been proposed. It can be used with this system. Many things can be performed with transportation systems in a smart city. For example, people can pay for transportation through a single interface and use a shared service simultaneously. When people and businesses use the intelligent transportation system app, it also helps them make smarter decisions about where they can go and how many cars are on the road. Another use for an intelligent transportation system in a city is tracking micro-mobility in the town. A system also tracks people so public transportation can run better. The main problems can be solved using the blockchain instead of a traditional database system, as shown in Figure 8.

### 4.5. Blockchain—Internet of Things for Banking and Finance

**Figure 9:**
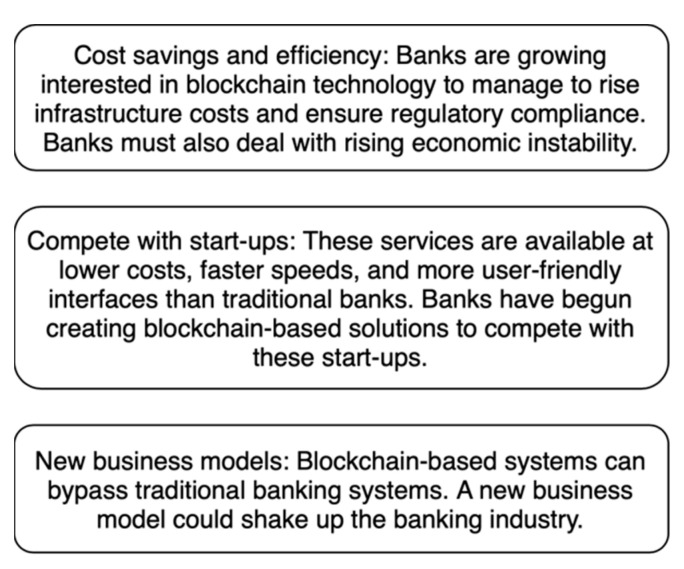
Main reasons for financial firms to invest in blockchain.

It is safe when people share information because it is stored on a “distributed ledger”. Decentralisation, immutability, efficiency, cost-effectiveness, and security are the main things that make people want to use blockchain technology in all financial services. People want to use technology because of these things. This means that the industry will change significantly in the next few years. It is a lot of work for banks to develop ways to cut down on the number of people who have to be involved in transactions. Banks worldwide have spent more time and money on big projects than other businesses [30]. Because of how blockchain works, some people want to invest in start-ups that use that kind of technology. Blockchain technology is used by many other businesses. Only a few people have started to patent their systems that use blockchain or the technology that makes them work. It is essential to make it easier for banks to conduct business and reduce the total settlement cost for blockchain companies. They should make sure legislators understand how these new solutions work to use them. Bank of America has filed many patents on blockchain to make and settle transactions. People from DBS and Standard Chartered Banks are working with Ripple on a trade-finance project to make tracking and avoiding double invoices easier. Figure 9 shows the main reasons financial institutions want to invest in and use blockchain technology.

### 4.6. Blockchain—Internet of Things for Smart Logistics and Customer Relationship Management

Performance-based supplier selection, logistics planning, and contract fulfilment are part of Smart Logistics, an all-in-one software package [31,32].

#### 4.6.1. Smart Contract

As long as the promises were written down in digital form and there were rules for how the parties could keep them, a “smart contract” was what Nick Szabo called these digital contracts. The intelligent contract module’s primary goal is to speed up writing a purchase contract. The module helps the buyer look for and choose the best supplier for their needs and lets them talk with the supplier about pricing and contract terms with the module’s help. To make a purchase order, a contract must be agreed to by both sides. Quality and delivery time are the primary obligations in the contract used as a source for the Condition Monitoring (CM) module, which is used to keep an eye on things and ensure things are going well.

##### Logistics Planner

There may be more than one person who can perform a job for just one person at a time, but they all work together. All suppliers may be in a different place and conduct more work. Several trucks move goods from the supplier’s warehouse to the customer. They come up with the best way to move the goods. When things go wrong with the plan, the planner is called in, such as when a truck breaks down and does not deliver the goods. Some parts of the programme may have to be changed. This means that the contract terms could also be broken.

##### Condition Monitoring

If the assets are always being watched, contracts must be maintained. That is why this part is so important. It can obtain data from all the sensors, controls, and other system parts that manage and change how the system works. There are times when the latency is significant for keeping assets safe, and this framework uses edge analytics. It is possible to make decisions based on what is happening through a distributed machine intelligence framework that tries to get around network bandwidth limitations and resource-constrained devices by making them work together.

#### 4.6.2. Functional Architecture

Our solution’s functional architecture shows how its parts work together. How will it work, and what are the most important features?

##### Smart Contract System (SCS)

**Figure 10:**
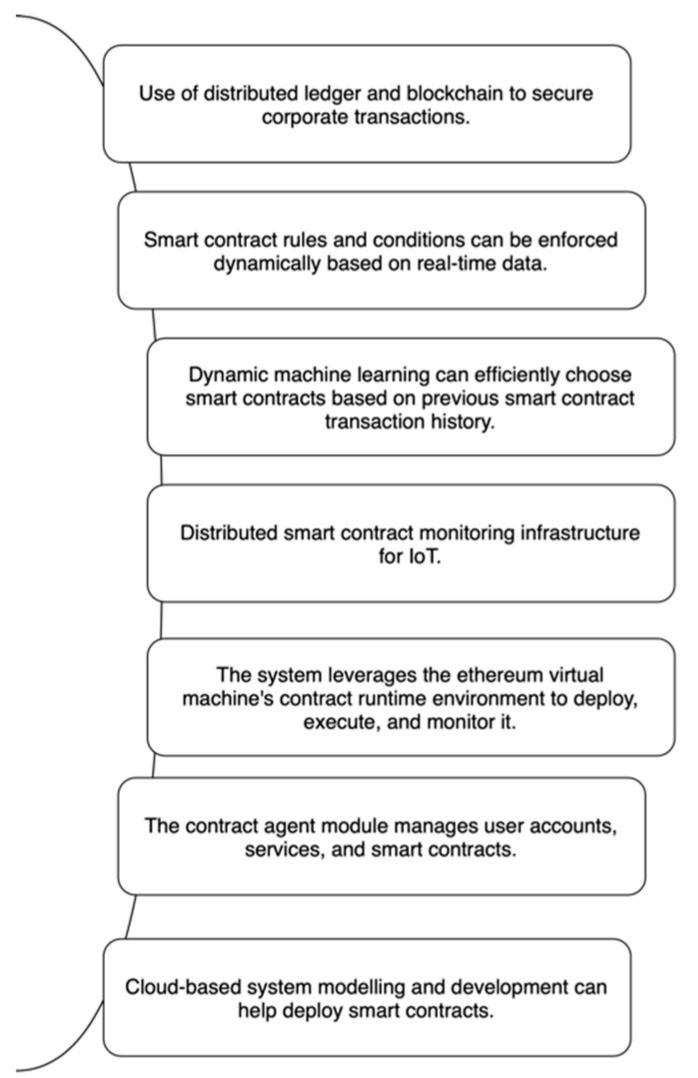
Key Features of Smart Contract System.

SCS has a list of people who sell a sure thing that needs to be bought. It then sends notifications to each supplier and talks about terms and conditions. There are a lot of essential things about the system shown in Figure 10.

After the negotiation process, a smart contract is made, and a purchase order is sent out to make the deal happen. The SCS also tells the planner and Condition Monitoring modules about the progress of the purchase order. The SCS checks to see if the contract terms are being kept. SCS takes the correct action if an agreement is broken, which could be a penalty or even cancel the order.

##### Logistics Planner

**Figure 11:**
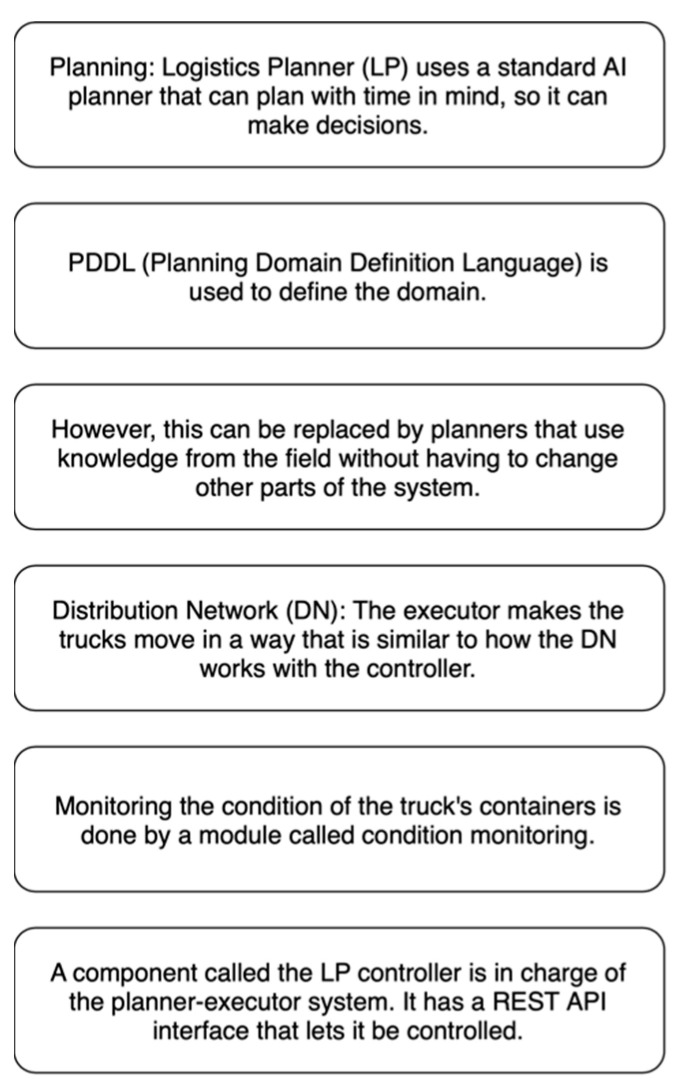
Key features of the Logistic Planner.

The Logistics Planner (LP) is in charge of developing and implementing the best plan to meet an intelligent contract. These parts are not very well-integrated, but they can be re-targeted to any other use case, and in many cases, they can be used immediately. A key feature of logistic planners is shown in Figure 11.

### 4.7. Blockchain—Internet of Things for Smart Energy

Electricity has been essential to our modern way of life for a long time now. During the last part of the 1800s, it became more common. It has made a lot of changes in the world of work, as well as a lot of changes in our culture [33,34]. This is because there is room for growth. Technology, such as the Internet of Things (IoT), blockchain, and other things, is changing the energy field significantly because of these new things. Energy production, system maintenance, power grid management, and home use could improve if these things were put together.

#### 4.7.1. In Energy Sector—Internet of Things

Some call IoT technologies the “Internet of Energy” (IoE) in the energy industry because of their work. It is also called “green technology.” Energy systems use a new term to discuss the technology that makes them more efficient and reduces waste. This is performed by putting a lot of intelligent sensors and devices in the parts of the power grid that need to be kept safe. People should remember that AI solutions, such as the ones above, could not have been made possible without IoT devices. A sensor is a piece of equipment used to collect data for AI-based improvement. Sensors are installed on production and distribution lines and end-user devices.

#### 4.7.2. Energy Sector—Blockchain

“Blockchain” might sound like a buzzword to some people or like a technology that cannot be used in the energy industry to others. The distributed ledger has a lot of potential in the energy field, which could be beneficial. Specifically, when looking at certain things, such as:

- Security concerns in energy networks: Energy providers are forced to use a blockchain because the energy grid is becoming more interconnected and digital, so they need to use one. The rise of IoT devices and AI-based solutions makes a lot of sense. Using the blockchain’s decentralised and encrypted ledger means providers can be sure that the data they are collecting cannot be stolen or changed in any way, even if someone else gets hold of it.
- Microgrid creation and implementation: Small microgrid networks connect end-users who are making their energy with solar and wind power and giving it back to the grid so that other people can use it. Blockchain might make it easier for people with extra energy to sell it and get paid faster and safer. These microgrids might use blockchain to make this easier. Some people do not like that all the power comes from one big company, and they want more people to use renewable energy instead.
- Renewable projects’ promotion: A second way that the blockchain could help the energy industry is by promoting environmentally friendly projects and making it easier for investors and entrepreneurs to get in touch with each other.

### 4.8. Blockchain—Internet of Things for Artificial Intelligence-Based Security

It is common to add internet connectivity, digital machines, objects, and people in the Internet of Things to each other because each device has a unique address and can automatically send data to the web, each one having its address. Even though they are not safe because they are connected to the Internet, they are not a good idea. IoT apps cannot be safe because they cannot be kept secure [35]. There are many ways to use blockchain technology, but the most common is using bitcoin, Ethereum, and smart contracts to control, manage, and protect IoT devices. So, there is a security risk when IoT apps use blockchain to connect. AI technology can be used to solve security problems, so use it. This technology solves the problem by taking care of data confidentiality, integrity, access control, authentication, non-repudiation, and much more.

- Data Confidentiality: As more and more people use IoT apps, they are being used in a wide range of fields. These include healthcare, transportation, weather forecasting, and so on. In healthcare, body sensors or physiological data allow billions of people who are disabled to go to medical facilities. These data are then sent to the medical staff to give the person medical attention. It is still essential for patients to keep their medical information private in IoT apps.
- Data Integrity: “Data integrity” refers to how well data are kept over IoT networks. It is good to have the best communication from one person to another. RSA, AES, and TDES algorithms keep this communication safe. However, the IoT has a problem with data integrity in IoT apps.
- Access Control: It is a way to ensure that only people who need the resources in IoT networks use them. Everything that has to do with what people do and how they do it is covered here. To solve this problem, blockchain technology is used. Because bitcoin can be quickly moved from one person to another, it can be performed promptly.
- Non-Repudiation: Non-repudiation means that any device on the Internet cannot say that a transaction was conducted from one person to another. This is because it already has authentication and integrity built in. These non-repudiation methods are used by IoT applications for security, such as digital certificates and hardware-based anchors of trust. Strong user non-repudiation should also control user access to IoT applications; it is not enough to protect all IoT applications.

### 4.9. Blockchain for Industry 4.0

**Figure 12:**
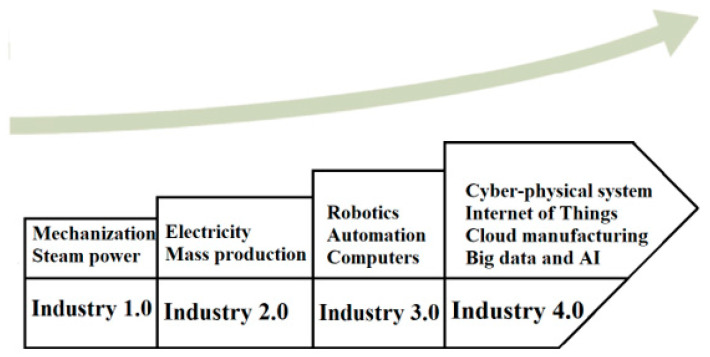
Historical Evolution of Industry 1.0 to Industry 4.0.

**Figure 13:**
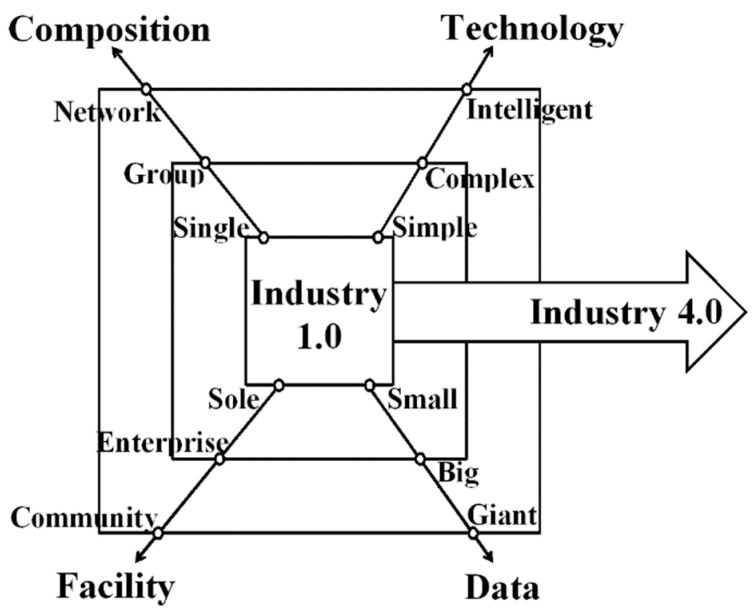
Characteristics of Industry 1.0 to 4.0.

**Figure 14:**
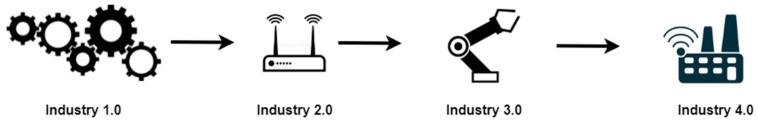
Industrial Evolution from Industry 1.0 to Industry 4.0.

People who work with technology say that the blockchain records all the money people have sent and received. The company owns the whole blockchain, except for the nodes that make it better than other ways to store data. Each node that took part in the transaction would see the ledger on many different things. People can trust and believe in the system because of this. In this way, there is a lot of network-wide autonomy and decentralisation. When people join a project, they first look at all the transactions that have been made on the blockchain. Blockchain technology, also known as distributed ledger technology, is a new way to store transaction information in a database that is not centralised and is easy for people to see. It is a new way to keep track of transactions. Nodes are the computers that make it work. This means there is no single point of failure, and information can be accessed in real-time [36]. The industry agrees with the idea that blockchain technology will change the market. It is shown in Figure 12, Figure 13 and Figure 14 how things have changed from Industry 1.0 to Industry 4.0.

Researchers can find detailed information about Industry 4.0 and Society 5.0 [1].

It has a trustworthy framework that marks all transactions and timescales in different processing stages, making it easy to trust. A new technology called blockchain lets businesses work with trust and test their faith, which is essential for companies. Blockchain is a type of technology. It became increasingly popular over time, and it helped the ecosystem in the long run. People make things that take a long time to go digital. It cannot keep ignoring the benefits of digitalisation, such as its efficiency, competitiveness, and speed. This is how it works: Data from sensors and machines can make things more efficient and save a lot of money. It will be even better with the help of blockchain technologies. The consumer goods industry is the most active in giving, making, and selling goods. There are smart contracts on the blockchain that make it easy for people to work together and make sure that everyone does what they say they will. There are a lot of counterfeits in the electronics, luxury, and lifestyle businesses because they do not have good traceability in all parts of the supply chain. Blockchain could be very good at stopping this because it could keep track of everything that goes into making a product. This is because more people are worried about the safety of connected cars being hacked. The digital revolution has had the most effect on manufacturing. Most businesses have changed now, but the disruption is more noticeable. Industry 4.0 undergoes many changes and expands the use of virtual data and processes.

### 4.10. Blockchain—Internet of Things for Society 5.0

New technologies such as the Internet of Things (IoT), AI, and robotics are made this time. These new technologies will significantly impact the economy and society [37]. It wants to make businesses more competitive and help people become more aware of their needs. Data, new technologies from the fourth industrial revolution, and how they can help solve social problems, such as a declining birth rate, an old population, and environmental and energy issues, are critical to many people.

Humans are the centre of Society 5.0, an ecosystem where people, IoT devices, and systems work together. Look at IoT data and send it back to the world with the help of AI! Unless problems with data monopoly, data abuse, and data ownership are solved, Society 5.0 cannot come to fruition. Google, Apple, Facebook, and Amazon are big companies with a lot of data. People are worried about data monopoly and abuse because these companies hold most of the data. Small- and medium-sized businesses cannot use data to develop new ideas because of a data monopoly. In the digital age, it is essential to share data with the rest of the world to be used to make things better. The use of data also needs to be clear about who owns it in order to be used well. When there are not any privacy issues, the person who made the data public should be in charge. It does not become dark. Everyone can keep transaction records, ownerships, and promises safe in a ledger called “blockchain,” which everyone can use. It is also possible to make digital smart contracts with blockchains, such as Ethereum, which is why they are so important. They can use these to carry out critical legal events or actions if they have a contract or agreement. They can also keep track of and record these things. It is not like a data monopoly because public blockchain data are open to everyone.

## 5. Open Issues, Challenges towards Blockchain—Internet of Things-Enabled Applications

This section discusses the main issues that need to be solved using blockchain technology in the IoT field. Using blockchain technology with the IoT is not easy. Blockchain was made for an Internet scenario where powerful computers were used [38,39,40]. The transactions on the blockchain are signed digitally, so devices that can work with the currency must do this. It is hard to put blockchain into the Internet of Things. Some of the problems that were found are shown in this section.

### 5.1. Storage Capacity and Scalability

Blockchain’s storage capacity and scalability are still up in the air, but these issues become much more difficult for IoT applications. Initially, it might seem that blockchain is not a good fit for IoT applications. However, there are ways to get around these limitations or avoid them altogether. IoT devices can produce gigabytes of data in real-time, making it hard for blockchain to work with them [41]. Currently, some blockchains can only process a few transactions per second, which could slow down the IoT. Because the IoT generates a lot of data, it does not make sense for blockchain to store it all. Integration of these technologies should be able to deal with these problems. Many IoT data have been held in the past, but only a few can learn and act. In the literature, different ways to filter, normalise, and compress IoT data so that they are less extensive have been proposed.

### 5.2. Security

People who write IoT applications have to deal with security issues on many levels, but also with the problems of low performance and many devices. Security is also affected by essential things such as mobility, wireless communication, or the size of the IoT scenario. An in-depth study of IoT security is out of this paper’s scope, but detailed surveys are. The more attacks on IoT networks and the severe consequences, the more critical it is to better security [42]. Many experts believe that blockchain is a crucial technology that can help improve the safety of IoT. When the IoT and blockchain are mixed, one of the main issues that need to be worked out is how reliable the data generated by the IoT are. Blockchain can ensure that the data in the chain cannot be changed. Still, if the data are corrupted in the blockchain, it stays that way. Many things can happen to IoT data that are not malicious. The well-being of the IoT architecture is affected by many things, including the environment, the people who use it, vandalism, and the failure of the things that use it. Sometimes the devices and their sensors and actuators do not work right from the start, and they do not work well.

### 5.3. Anonymity and Data Privacy

For example, when a device is linked to a person, such as in the case of eHealth, it is essential to consider how to protect data privacy and anonymity. Blockchain is the best way to deal with identity management in IoT, but obscurity is critical in many applications. This is the case of a wearable that can hide the person’s identity when sending personal data or smart cars that protect the users’ routes. Data privacy in transparent and public blockchains has already been discussed, as some of the existing solutions to this problem and how they might work. However, the issue of data privacy in IoT devices is more difficult because it starts with data collection and goes up to communication and applications. Many steps must be taken to ensure that the device is safe and that people can only access the data they are supposed to. This includes installing cryptographic security software into the machine. These changes should consider the limited resources of the devices and the limits on how profitable they can be. Many different technologies have been used to protect communications with encryptions.

### 5.4. Smart Contracts

Smart contracts have been called the “killer app” for blockchain technology, but many problems still need to be solved. In the IoT, smart contracts could be helpful. However, how they work in IoT applications is different. A warranty is a piece of code and data stored on a specific blockchain address when it comes down to it. Public functions in an agreement can be called by other things, such as phones or tablets. As well as firing events, functions can also send out notifications. Applications can listen to these notifications to respond to the event correctly. It takes a transaction to make changes to the contract, which means making changes to the blockchain so that the contract changes.

For a transaction to go through, the sender must sign it and the network must accept it. The Internet of Things can sense and act over the Internet in many places. Food packaging has sensors to measure the environment and connect to the blockchain in the food traceability example. This way, the food could be traced back to where it came from (sign transactions). A contract in the blockchain could start and finish shipping and log and look up measurements [43]. An event would be triggered if a certain number of measures went above a certain level. When these events happen, management applications will be listening to them. The shippers and retailers will be told. Manufacturers will also be notified. If there were no problems with the shipment, the blockchain would ensure that it was performed in the best way possible. It would be safe and reliable if intelligent contracts were used to process the IoT, recording and managing its interactions. If the processing were safe and dependable, the actions would follow. So, smart contracts can be used to safely model the application logic of IoT applications. However, some issues need to be worked out in that integration. When researchers work with smart contracts, the researcher needs to use oracles, unique entities that give real-world data in a trusted way. Validating these smart contracts could be harmed because the Internet of Things (IoT) is not always stable.

### 5.5. Legal Issues

The idea of a blockchain that is not regulated is part of what makes bitcoin so great. Blockchain, especially when it comes to virtual currencies, has caused a lot of debate about whether or not it is legal. The need or chance to add control elements to the network has come from permissioned, private, and consortium-based blockchains. The laws and regulations that deal with data privacy also impact the IoT field, such as the data protection directive. Most of these laws are becoming out-of-date and need to be changed, mainly since new disruptive technologies, such as blockchain, have emerged. The development of new laws and standards can make it easier to certify IoT devices’ security features. This can help build the most secure and trusted network. When IoT and blockchain are used together, the laws dealing with information privacy and handling are still a big problem.

### 5.6. Consensus

It is not suitable for IoT applications for devices to directly participate in consensus mechanisms, such as PoW, because they do not have enough resources. There are a lot of different ideas for consensus protocols, but in general, they are not very mature and have not been tested enough. The consensus protocol used in a blockchain network affects how much space and money is needed. Gateways, or any other device that does not have to be limited, are usually used to do these things. Optionally, off-chain solutions, which move information outside of the blockchain to cut down on the high latency in blockchain, could be used to perform the job. Even though there are projects to put full blockchain nodes on IoT devices, mining is still a big problem because of its limitations.

### 5.7. Scalability

There are some problems with current blockchain platforms. This means they are not very scalable, have limited throughput, and cost a lot to run. Many blockchains have extended processing times for transactions to be added to the blockchain that have already been confirmed. This is because many blockchains have a limited block size. As a result, the block time proliferates, slowing down the system. As time goes on, the ledger will get very big. Thus, the amount of IoT data would increase, making it difficult to process large amounts of data in the blockchain. Because of these problems, many app developers do not see blockchain technology as a suitable replacement for existing systems for large IoT systems.

### 5.8. High Computational Cost

The process of processing a transaction includes many steps, such as setting up a lot of security, mining, validating, and storing it across many people. Among other things, Proof of Work (PoW), the most decentralised way to mine, solves a complicated math problem that requires a lot of powerful computer hardware. PoW needs a lot of resources to qualify as the most decentralised, and IoT systems do not have enough of them. The complexity of the blockchain system will require a lot of technical and human resources. This would make people worry about the high costs of running blockchain-based systems, which would keep them from using them on a large scale.

### 5.9. Trust Issues Depend on Security and Privacy

On the other hand, blockchain can withstand major security attacks such as Sybil, Distributed Denial of Services (DDoS), selfish mining, Ransomware, and more. On the other hand, the existing blockchain has some trust issues directly dependent on security/privacy attacks. Blockchain machines can change consensus processes and stop new transactions from being approved if more than half of them can control computing resources. This means that they can do this for the wrong reasons. People who do not keep a close eye on transactions could put the blockchain at risk of losing information and disrupting the network, resulting in a loss of trust. In a Sybil attack, the malicious nodes make up a lot of different identities so that they can flood the network with transactions or make up stories about traffic jams, for example. DDoS attacks are brutal to perform on a network of blockchains. DDoS attacks and these attacks are the most common on blockchain networks. Such attacks can shut down a network and harm users’ personal information, again causing a loss of trust. Note that blockchain technology can be hacked, and these attacks are the most common on blockchain networks. DDoS attackers try to stop the mining process, e-wallets, crypto exchanges, and other financial services on the web. Selfish mining is a strategy for mining bitcoins in which groups of people work together to make more money.

## 6. Open Issues, Challenges, and Future Research Opportunities towards Implementing Blockchain in Industry 4.0 and Society 5.0

In the past decade, several open issues, technical, legal, etc., have been found in implementing blockchain in Industry 4.0 and Society 5.0. Such issues and challenges may be mitigated shortly after implementing blockchain in innovative applications [44,45]. Hence, such scenarios are explained below.

### 6.1. Open Issues towards Implementing Blockchain in Industry 4.0 and Society 5.0

It is essential to have real-time data in the Industry 4.0 environment to ensure smooth manufacturing and service systems. Processing time is the main problem, so careful thought must be given to real-world applications. Keeping track of records and information is accessible with blockchain. It can also deal with a lot of big problems. As a result, this study aims to figure out how blockchain works in Industry 4.0. In the corporate world, it is time to start using blockchain again. This will make it easier for people to conduct business. People who make things in Industry 4.0 use blockchain technology to make them more environmentally friendly [46]. This makes it easier to keep track of the products that people make. This intelligent factory with cutting-edge technology could help keep information safe. This will be able to thrive in production environments that are less risky and give a better level of process safety. However, before businesses use blockchain, a lot of work with the platform must be performed. The risk level can be reduced, and because technology is improving, business leaders must know how technology can help their companies. Industry 4.0 calls for more trust and privacy. 

It is called “Society 5.0”, a slow-moving revolution that started in Japan. It promises to change society by putting people at the centre of technological and innovative changes for all good. “Society 5.0” is when AI ideas from the past are used to build new Cyber-Physical-Social systems (CPSs). It has become a big part of how businesses and society have changed over the last few years. Parallel intelligence can help people and businesses deal with complex social and engineering problems. It can also help companies. The goal is to develop quick, targeted, and convergent ways to understand ambiguous, diverse, and complex issues. Global warming, terrorism, and a widening gap in the economy are all problems that the world has to deal with. People in today’s world live in a new era of technological progress, the era of virtual–real intelligence technology. The reason for this is that there have been a lot of changes in mechanisation, electricity, information, and networking technology. Connecting people and things and the real and virtual worlds will result in many good things. In the long run, this will help people have a better life and keep the economy growing at a healthy rate. There is a new structure for managing and controlling CPSs as AI technology improves. In an intelligent society, digital technology, instruments, and networks are used to improve life. It can connect different industries, countries, regions, and societies. Note that CPSs can do this. Three things make up ACP: artificial systems, computer experiments, and parallel execution. Society 5.0 came to existence when the information society began to grow and change. Businesses and people could also benefit from it, and it tried to find ways to solve problems in the world.

### 6.2. Challenges towards Implementing Blockchain in Industry 4.0 and Society 5.0

This technology can store all the data digitally to make businesses run faster and better. A company can only use new technology and make money if it has the right staff. This technology can be used in the right way to solve problems with data privacy, and it can help. People who work for many different businesses can share data and make it easier to share data. Even if there are problems now or shortly with how things are made, that is a big deal. They can talk to each other in real-time on an identical blockchain. This can connect to one or more networks. Those pieces of equipment could have flaws, which would leave them open to attack. Industry 4.0 can help with a lot of different types of security.

Most modern devices will connect to the Internet, automatically sharing data and interacting with each other. This means that all “things” that people interact with at home, at work, and in production and use will be interactive in new ways. This is the main difference between Society 5.0 (information society) and the previous Society 4.0 (computer society), which was only connected to “computers”. To make things even better, all the data will have to be put together as big data outside the individual servers, and AI will have to make real-time suggestions. This will require a lot of data to be used. In today’s business world, there are a lot of different data silos.

Big data are being used so that other people have already done it. People do not talk to each other about things like this because they work for different companies. Micropayment for such data is not used. If these problems are not solved, it will be hard to acquire Society 5.0.

There could be less innovation and improper use of our data if a single business owned all of our data. Our privacy and freedom of speech could be sacrificed to make more money. There are two main reasons why this is the way it is. One is that the Internet platform is very centralised. Because an individual cannot be sure of their identity on the Internet now, centralised control is needed to ensure data are consistent and secure. On the other hand, our capitalist society makes sense for businesses with a lot of data to use to make their businesses more valuable.

It is also because the data are not easy to move around. Data are rarely sold to another company because they are a barrier for other businesses. However, capitalism’s basic rules say companies should make more money for themselves. There is not enough money for small businesses to obtain more data, so big companies with a lot of money end up with too much data. People who think that data are a barrier to entry only go into areas where they can make the most money and do not care about social issues. This is why, in my opinion, blockchain technology will be beneficial.

### 6.3. Future Research Opportunities towards Implementing Blockchain in Industry 4.0 and Society 5.0

Blockchain will be suitable for commercial finance, supply chain activities, operations management, and other related things. Many disagreements could be solved quickly if they were made to fit each person. This will reduce the manual work that financial service companies have to do now. If there are any inconsistencies or critical flaws in the text, they will be found by the AI process, which will help people decide whether to accept or reject the text. Many groups are trying to get people to connect the IoT and blockchain. This firm has developed a way to combine linked devices with safe, accurate digital data archives. Blockchains are being used by businesses to help them run better. This technology will store and share data in the same way. People can track where products are seen when blockchain is used in real-time. This will make the trail of goods smarter and more secure. Each transaction in the supply chain process is kept on the blockchain, which gives a complete picture and long-term information [47]. To make the supply chain more efficient, it will connect it with all the people and places it needs to get things to. Blockchain will change business practices in a wide range of industries in the future, but it will take time and effort to get businesses to use it. It will help improve financial and public services shortly with the help of this technology, too. Data are stored in blocks that cannot be changed. Each block is linked to the next and has a timestamp. If a product is new or used, users will see its history thanks to blockchain. In short, this type of blockchain application could help cut down on inconsistencies between planning and execution in the Industry 4.0 world. This connection is essential for developing Industry 4.0, and academics must pay attention to their problems. So, policymakers and managers can use blockchain to move towards Industry 4.0; they need to deal with the drivers, enablers, and constraints that have been identified. Data query processing, on-chain data storage, hybrid data storage, and cloud are some of the major fields that can be further researched regarding the current area of focus.

People’s jobs, government administrations, their privacy, and the economy’s structure are all changing a lot, and digital information needs to change to meet these new needs. To make Society 5.0 a reality, it must consider many different things, such as entrepreneurial skills, entrepreneurial spirit, and innovation policy. The use of new technology can help people live better lives, but it can also hurt jobs, wealth distribution, and information. Society 5.0 allows modern technologies, such as IT, IoT, robotics, artificial intelligence, and augmented reality, to be used in people’s daily lives, health, and other areas of their lives, while Industry 4.0 only allows new technologies to be used in factories. Economic growth and social problems could be solved with more production, less food, and other technologies for the good of humanity. These technologies could help solve social issues and make it easier for people to acquire what they need. Using the power of technology and the creativity of many people, Society 5.0 can meet the United Nations’ sustainable development goals.

## 7. Research Statements for Blockchain and Blockchain—Internet of Things-Enabled Applications

Note that today’s researcher looks at the most critical parts of blockchain technology and its characteristics with this background. Many people have been interested in innovative home systems in the last decade because they make people more comfortable and improve their lives. Sensors and actuators that have intelligence can be used to make smart homes. The researcher could use this method to send data and services quickly and keep them safe. Many people may need to use many smart home devices simultaneously in a smart home. In the Internet of Things, data can be processed and exchanged without the help of people. They must recognise, verify, and keep the integrity of their communication data to be in complete control. An IoT device may change a smart contract as a product moves from the manufacturer to the store. This section discusses some crucial open research problems preventing blockchain technology in a smart city. The researcher explains what caused these problems and how to deal with them [48,49,50].

### 7.1. Sustainability

Sustainability refers to the design of blockchain-enabled smart cities that do not use too many environmental resources. Smart cities use a lot of energy because there are a lot of intelligent IoT devices. In addition, blockchain consensus algorithms such as PoS, which have a lot of computational power, also increase energy use. Many factors must be taken into account to make a smart city that is environmentally friendly and uses blockchain technology. These factors include energy-efficient communication networks, renewable energy resources, energy-efficient storage for blockchain, energy-efficient consensus algorithms, and reputation-based consensus schemes. There are a lot of energy-efficient consensus algorithms out there, such as PBFT, PoS, DPoS, proof of activity, evidence of importance, and proof of retrievability, which all work together to keep energy consumption down the most. Similarly, hardware that does not use a lot of energy, such as Application-Specific Integrated Circuits (ASICs) for PoS-based blockchain, can be used for innovative city services that are environmentally friendly. More energy-efficient validation schemes need to be made to make blockchain-based innovative city services long-term sustainable from an economic, environmental, and social point of view.

### 7.2. Adaptive Consensus Algorithm

Innovative city improvements have a lot of different things that need to be considered when the are made. So, there is much room for research into making the smart city more flexible using the blockchain. A consensus algorithm gives rules for reaching a consensus with other nodes in a blockchain network in a typical blockchain. Every blockchain consensus algorithm has a unique design based on the node’s identity, energy consumption, data model, and use. PoW has a lot of energy use, a public node identity, a transaction- and account-based model, and a cryptocurrency application.

Similarly, PoS has a part that saves energy, a public node ID, and an account-based model. For example, suppose energy efficiency is one of the main goals of blockchain-enabled innovative city advancements. Different consensus algorithms can be used for the same applications, but with varying amounts of energy. PoW and proof of activity use the same data model and cryptocurrency application. Still, they use different amounts of energy to do so (i.e., evidence of activity has lower energy consumption). So, the researchers need to make a consensus algorithm that can change with the application’s needs. The number of validators changes based on game theory in their consensus algorithm. There is less chance of going wrong when researchers pick only a valid number of honest validators.

On the other hand, an algorithm uses artificial intelligence to combine the benefits of DPoS, PoS, and PoW into a single algorithm. The authors discussed blockchain type, adversary tolerance, scalability, communication model, throughput, bandwidth, and consensus finality. However, more research is needed to figure out how to design and implement consensus algorithms like this.

### 7.3. Scalability

Scalability refers to how innovative environments made possible by blockchain work as smart city devices grow. It is also essential for a typical blockchain network to deal with problems and be secure. However, achieving these features simultaneously limits the city’s growth, one of the most critical factors in clever city design. Every full node in a blockchain network needs to store more and more records and be a part of the validation process. It is hard to scale because a typical blockchain is entirely decentralised. Many smart IoT devices present problems in designing scalable blockchain-enabled smart city infrastructure. So, to make the innovative city services that use blockchains more scalable, the researcher needs to develop consensus algorithms with consistency, availability, and partition tolerance. Many ways have been suggested to make blockchain more scalable. Some blocks were thought to reach consensus, such as checkpoint blocks, rather than all transactions. PoW is a consensus algorithm that tries to keep a blockchain network as secure and stable as possible. However, the traditional PoW protocol significantly impacts the scalability of blockchain in terms of how many transactions per second it can handle. To solve the scalability problems of the conventional PoW protocol, a PoW based on parallel mining is being proposed for the blockchain network to deal with them. That is why this solution is not the best one. The manager node has one point of failure.

### 7.4. Latency

Latency is the time it takes for a transaction to be processed. Throughput is the maximum number of transactions performed in a specific time. Both latency and throughput constraints significantly impact how big smart cities can be. There is a delay in a blockchain-enabled smart city because of work performed by computers that are not all in the same place. There was also a lot of communication between nodes that caused even more delay. Two things affect the latency and throughput of the blockchain network. Many solutions must be found to make smart cities possible with low latency and high throughput on the blockchain. A delay in propagation causes forking. For example, if a miner is successful, they send a block to a network. Some miners broadcast their blocks before they obtain the blocks from other miners. This effect causes miners to own more than one block at a time. The propagation delay must be as short as possible to avoid the forking effect. A scheme called “Closest Neighbors Selecting” (CNS) helps speed up information in the blockchain network. Following the forking, the process of making new blocks starts again. This process continues until the block update happens without splitting it into two. The forking effect makes it more likely that transactions will be confirmed because there is a chance that a long chain will not include the relevant block in the future, so there is a chance that the transaction could be rolled back. For example, the researcher should wait for at least six block confirmations in bitcoin, which adds up to an hour. It takes advantage of the upper limit on the time it takes for a PoS-based blockchain to keep from forking. People who use an intelligent city service based on a blockchain can use the ACCEL.

### 7.5. High-Performance Computing Memories and Storage

In short, smart IoT devices will be more common in smart cities. These devices have a lot of storage space, making it hard to provide blockchain-based smart services. Every device in a blockchain network must record all the transactions that have happened—having blockchain nodes that need a lot of storage space limits how big the network can get. On the other hand, scalability is one of the essential things for smart cities. To run blockchain-enabled smart cities at a large scale, high-performance computing memories with a lot of storage space and low power consumption are needed. This raises security and reliability concerns. Blockchain-based smart city services cannot work if the centralised memory does not work. Another problem with external memory is that it costs more and takes more time to manage. Instead of having centralised storage, some blockchains may use off-chain decentralised storage and file systems, such as IPFS and Swarm, which are not on the blockchain. However, IPFS and Swarm are open to the public, making it challenging to use them for private data. However, data can be encrypted before uploading to IPFS, but this adds more time to the encryption–decryption process. As a side note, sharing encryption–decryption keys in a way that is not centralised but safe is another problem.

### 7.6. Secure Economic Models

Future smart cities are expected to use 5G and beyond telecommunication networks and new computing paradigms to provide a wide range of smart services with a wide range of needs. Network slicing is an excellent way to let these innovative services work with changing conditions. This is how network slices work: Physical networks are sliced into logical networks that can be used on top of them. The network slicing operator must buy different physical resources from service providers and then sell them to smart people who want them. Network slicing in smart cities requires new and safe economic models that improve the user’s experience and the service provider’s profit. There are many ways to make money for people working in a smart city. However, the researcher needs to develop new ways to make intelligent city services profitable to meet a wide range of customer needs. These applications and user expectations include latency, operational efficiency, privacy, security, service provider profit, and user quality of experience, to name a few. Network slicing can provide a wide range of services for smart cities. Blockchain-based brokers buy resources from many resource providers and safely sell them to smart people from other industries.

### 7.7. Identity and Privacy

In public blockchains, everyone can see all of the transactions that have been made so far. Each device part of the network can be identified by its public address. Even though the public discourse is a pseudonym, curious malicious actors with some background information can use the links between public addresses and transactions to discover the real-world identity of people who use them. People who use cryptocurrency smart city applications can make each payment to a new, disposable address. They can also use mixers that collect and redistribute coins to the right people. The user’s identity in smart city services is currently provided by digital identity management systems run by central authorities. Self-Sovereign Identity (SSI) and Decentralised ID (DID) let people control their digital identities without working with a third party they do not own. This allows people to decide how their personally identifiable information and data are shared. A blockchain-enabled SSI and DID can be used in smart city services to identify, authenticate, and authorise users in a way that a single person or group does not control. Creating safe ways to get back into SSI and DID is a big problem. As a result of the public blockchain being open to everyone, users’ data and pseudonymous identities can be accessed. This raises privacy and identity threats. Privacy methods, such as zero-knowledge proofs-based distributed consent management and double-blind data sharing, can be used to share only certain types of data with other people in multi-party transactions that are both anonymous and private in smart city services. However, symmetric on-chain encryption and different kinds of encryptions may be used to protect the transaction data themselves. One of the drawbacks is that these techniques add more time to the network.

### 7.8. Smart Contract Immutability and Chain-Boundedness

Smart contracts are contracts that cannot be changed. This helps to build trust between the parties of the contract. In most cases, though, the smart contract code (e.g., on the Ethereum Platform) cannot be changed even if there are bugs, vulnerabilities, or new business rules. The updated code for the smart contract is usually put into place in a new instance with a new contract address, which could cause problems. The new contract address could be different from the old one. However, there is a way around this for a smart contract upgrade. The proxy contract holds the data while the logic contract does the new logic, which is why it is called a proxy contract. Each time the proxy contract is updated, the logic contract address is changed. Smart city service users will not be affected by the update because their data are safe in a proxy contract, so they will not have to change anything. Trust and decentralisation issues are still problematic when using a proxy to call or contact someone else. Researchers are coming up with ways to make a smart contract that is not wholly upgradable. They do not allow changes to the core functions of a smart contract, but some parts can still be changed. Another problem with the smart contract is chain-boundedness, which means there is no way to request things outside of itself. The only way the intelligent contract interacts with real-world deterministic information is through event-triggered oracle data feeds for deterministic information. However, there is still a lot of work on the decentralisation, determinism, authenticity, trust, and security of oracles.

Finally, other uses of blockchain can be found in different applications (including several critical issues, challenges, etc.) in [51,52,53].

## 8. Conclusions

Blockchain, called a game-changer technology, is getting more attention from research and the business world. The attention this technology is getting in the media may make it hard to make an objective decision about whether or not to invest in it. Businesses risk adopting blockchain technology because they are curious about it, not because they think it is ready for use in companies and the general public. Ultimately, they developed some new architecture that improves security and data transparency. Blockchain-based smart agriculture uses the unified smart home resource services; therefore, users must be able to prove that they are who they say they are using blockchain technology’s smart contract idea. The most important thing about this work is that services are used more quickly and safely. Because the smart home resource cannot be used by anyone else, repeated authentication does not need to be performed. Intelligent cities want to improve the quality of life for their residents by giving them innovative and sophisticated services. It is important to point out that people’s different ways of gathering, storing, processing, and analysing data can make them vulnerable. People may be able to get their hands on smart city data and apps in the future because of things such as the Internet of Things (IoT), cloud computing, social media, and more. This flaw could put the safety of sensitive data at risk, so it needs to be fixed. Blockchain technology is being used in the IoT, healthcare, and finance, to name a few. New markets are being opened because of the Internet of Things (IoT). Companies are also getting a head start in their current and new needs.

Financial data are just as crucial because of threats, such as man-in-the-middle and DoS, when predicting future market trends and storing clients’ investment details. The use of blockchain technology can also help with this problem. Blockchain technology can change how healthcare is conducted by putting patients at the heart of the system and improving data security, privacy, and interoperability. Blockchain-driven AI solves the problem of blockchain using AI. It is also important to note that the researcher talked about a high-level taxonomy of AI for blockchain in IoT and blockchain for AI in IoT, including topics such as AI-driven and blockchain-driven AI and a more detailed classification of blockchain-driven AI for IoT. Incorporating all three technologies will strengthen and help the technology’s weak points.

## Figures and Tables

**Table 1:** Comparison of Current Works in the Field.

Citation	Work
[10]	The principles of Industry 4.0 have been examined in this study. Many descriptions have changed, stressing an individualisation paradigm, even if they were originally summarised using its nine-pillar system.
[11]	Blockchain protects intellectual property rights, certification, and long-term accountability for replication in additive manufacturing.
[12]	This document provides insight into the idea, framework, and effects of implementing Society 5.0 from a sustainability perspective. To address the issues of contemporary society and advance the potential of individual–technology interaction, Society 5.0 suggests that sustainable digital innovation be used to create a super-intelligent society that would improve people’s quality of life.
[13]	This study, based on a literature assessment, tries to summarise the crucial role of AI in implementing Industry 4.0. The study objectives are, therefore, designed to aid in this paper’s accessibility to researchers, practitioners, students, and business professionals.
[14]	This work has discussed how the supervision of accounting education has changed due to the Industrial Revolution 4.0 and Society 5.0. Examining the strategic environment, problems, and dangers is appropriate for ongoing growth. Thus, it can be said that the accounting profession has partially anticipated the Industrial Revolution 4.0 and the advent of the Society 5.0 age. However, considering all the problems and dangers that have been recognised, there is still much potential for development.
[15]	Forty-one publications pertaining to the topic thus suggested could be found in the systematic literature review. Nineteen (19) terms were grouped to constitute the four (4) primary constructs imagined and explored by the analysis of these articles using the VOSviewer software: Industry Strategy, Innovation and Technologies, Society, and Issues of Sustainability and Transition.
[16]	This work uses text mining with unsupervised machine learning techniques to examine 3901 news stories and 660 academic articles to comprehend and clarify Industry 4.0. Based on the findings, this article outlines 31 research and application challenges connected to Industry 4.0.
[17]	The authors’ goal in conducting this systematic literature evaluation is to examine applications in many important business processes. Additionally, it outlines the main difficulties in using blockchain technology and its potential for corporate management. Both practitioners and academics worldwide.
[18]	This essay tries to analyse the obstacles to social inclusion that both historical and contemporary prospective social disparities present. This study employed a qualitative technique. The authors used the content analysis method to scan multiple international databases for papers on the study’s topic. The findings allow concluding that, while it is certain that the concept of Society 5.0 initially had a Japanese national dimension, it tends to be applied by those parts of the world that seek future sustainable development, with modifications taking into account the unique features of several countries (economic, social, and environmental).
[19]	In this article, the authors discuss the benefits and drawbacks of agricultural digitalisation using the artificial trolley dilemma as a framework. In this situation, one must decide whether it is moral to put someone at risk to prevent some clear and impending negative effects on a larger group of people. They also emphasise the requirement for new digital agricultural revolution trajectories that guarantee an increase in food production without having significant adverse social effects.
[20]	In the context of 5G and beyond, this article offers a brief and concise overview of the most recent research on blockchain-based decentralising applications. They explain five areas of motivation for decentralising apps using blockchain and offer four urgent 5G and beyond problems.

## Data Availability

Not applicable.
